# Comparative efficacy of a new spot-on combination product containing selamectin and sarolaner (Stronghold®Plus) *versus* fluralaner (Bravecto®) against induced infestations with *Ixodes ricinus* ticks on cats

**DOI:** 10.1186/s13071-017-2259-5

**Published:** 2017-06-29

**Authors:** Thomas Geurden, Stasia Borowski, Magda Wozniakiewicz, Vickie King, Josephus Fourie, Julian Liebenberg

**Affiliations:** 1 Zoetis, Veterinary Medicine Research & Development, Mercuriusstraat 20, 1930 Zaventem, Belgium; 2 Zoetis, Veterinary Medicine Research & Development, Portage Road 333, Kalamazoo, MI 49007 USA; 3 Clinvet, PO Box 11186, Universitas Bloemfontein, 9321 South Africa

**Keywords:** *Ixodes ricinus*, Cats, Efficacy, Persistent, Application, Sarolaner, Selamectin, Fluralaner

## Abstract

**Background:**

Ticks are increasingly reported on cats worldwide, with *Ixodes ricinus* being a relevant species across Europe and in near by areas of North Africa and the Middle East. Yet there are few acaracidal products with proven efficacy approved for use in cats. The objective of this study was to compare the efficacy of a new spot-on formulation containing selamectin and sarolaner with a topical application of fluralaner (Bravecto®) against *Ixodes ricinus* ticks on cats. To that end, twenty-four (24) cats were randomly allocated to one of three treatment groups. The cats in the control group remained untreated. Cats in group 2 were treated with selamectin/sarolaner (Stronghold®Plus; Zoetis) at the minimum recommended dose of 1.0 mg/kg sarolaner and 6.0 mg/kg selamectin on Days 0, 30 and 60. The cats in group 3 received a fluralaner treatment (Bravecto®spot-on solution for cats, MSD) at the minimum recommended dose of 40.0 mg/kg on Day 0. Cats were infested with 50 (± 4) viable, adult, unfed *I. ricinus* ticks on Days 26, 54, 82 and 89 and ticks were removed for counting 48 h (± 2 h) later.

**Results:**

Three monthly treatments with selamectin/sarolaner provided high and consistent efficacy against *I. ricinus* for the entire duration of the study period. In contrast, the efficacy of fluralaner declined in the second month after treatment and was below the efficacy threshold of 90% on Days 56, 84 and 91. The percentage efficacy against *I. ricinus* was numerically higher in the selemectin/sarolaner treated group than in the fluralaner-treated group on Days 56, 84 and 91. Furthermore, greasiness and spiking of the hair, as well as white deposits were frequently observed in the fluralaner-treated cats.

**Conclusion:**

The results of the present study confirm the high and consistent efficacy of a new spot-on combination product containing selamectin and sarolaner against *I. ricinus* in cats, and indicate a decline in fluralaner efficacy during the 91 day period after treatment.

## Background

Ticks are increasingly reported worldwide with *Ixodes ricinus* (the castor bean or sheep tick) known to be prevalent in cats in Europe, next to *Dermacentor reticulatus*, *Rhipicephalus sanguineus* and *Ixodes hexagonus*. [1–4]. Tick infestations can cause anemia, tick toxicity, papular dermatitis and alopecia, or lead to the transmission of vector-borne diseases [5]. In cats, treatment and prevention of tick infestations mainly relies on the use of acaricidal drugs, most of which either have a persistent efficacy less than four weeks or efficacy against a limited range of tick species. Recently, fluralaner (Bravecto® topical; MSD) has been approved for use in cats, for the treatment and prevention of *Ctenocephalides felis* and *Ixodes ricinus* for 84 days or 12 weeks.

In dogs, the isoxazoline class drugs are increasingly used in the treatment and prevention of ectoparasites, and sarolaner (Simparica®, Zoetis) has been distinguished among members of the class for its persistent efficacy for the full monthly dosing period and spectrum of ectoparasites on the label. Due to its excellent activity against ectoparasites [6] sarolaner has been combined with selamectin in a new spot-on formulation (Stronghold®Plus; Zoetis) to provide a broad efficacy spectrum. Previously it was demonstrated that treatment with selamectin/sarolaner provides month-long protection against all relevant tick species in cats [7]. The objective of the present study was to evaluate the efficacy of three monthly treatments with selamectin/sarolaner against *Ixodes ricinus* on cats and to compare the persistent efficacy of a single treatment with fluralaner.

## Methods

Twenty-eight (28) domestic shorthair cats were acclimatised to study housing seven days prior to the first treatment on Day 0. All cats were housed individually in cages and no physical contact between cats was possible. However, animals had visual and auditory contact with conspecifics. The floor size of each cat cage was 1.35 × 1.04 m. The study followed a randomised block design based on host-suitability tick counts performed during the acclimatisation period. The cats were ranked in descending order of host-suitability tick counts and subsequently blocked into eight blocks of three cats. A total of 24 cats (14 males and 10 females) were enrolled in the study. Within blocks, cats were randomly allocated to one of the three treatment groups with 8 cats per group. The experimental unit was the individual cat. Blinding was maintained by coding the treatment groups and by separating out study activities throughout the study.

The cats were weighed on Days -2, 29 and 58 to calculate the dosing. Cats in group 1 (control group) remained untreated. On Day 0, the cats in group 3 were treated with fluralaner (Bravecto®; MSD) at the minimum recommended label dose of 40.0 mg per kg body weight. On Days 0, 30 and 60, the cats in group 2 were treated with the new spot-on formulation (Stronghold®Plus; Zoetis) at the minimum recommended label dose of 1.0 mg/kg sarolaner and 6.0 mg/kg selamectin. During treatment administration disposable gloves and aprons were worn to avoid cross-contamination. The disposable protective clothing was changed between animals. The table on which the cat was placed was cleaned with a disinfectant cleanser between animals. The dose was applied through parting of the hair until the skin was visible and was applied topically at one spot between the shoulder blades. Cats were restrained for approximately one minute following treatment administration, to ensure complete application of the full dose. The cats were observed at different time points after treatment for possible adverse reactions to treatment. On Day 0, administration site observations were performed on all cats 30 min (± 5 min), 3 h (± 15 min) and 24 h (± 1 h) and again on Days 3 and 5 after treatment. Spiking of the hair (stiff hair), greasiness, white deposits or any other observation were recorded. Administration site observations were not performed on Days 30 and 60.

On Days 26, 54, 82 and 89, all cats were infested with approximately 50 (± 4) viable, adult, unfed *I. ricinus* ticks (1:1 sex ratio). To facilitate tick attachment, cats were sedated and placed into an infestation chamber for up to four hours following infestation. In addition, each cat was fitted with an Elizabethan collar from the time of infestation until the time of tick removal. Ticks were counted in situ (thumb counts without removal of the ticks) on Days 27, 55, 83 and 90 (24 h ± 1 h after infestation). Ticks were counted, removed and categorized on Days 28, 56, 84 and 91 (48 h ± 2 h after infestation).

Efficacy was calculated based on arithmetic and geometric mean tick counts, as follows: 100 × (M_c_ – M_t_)/M_c_, where M_c_ is the mean number of live ticks on cats in the negative control group (group 1) at each time point and M_t._ is the mean number of live ticks on cats in the respective treatment groups (groups 2 or 3) at each time point. Treatment groups were compared using a general linear mixed repeated measures model (Proc MIXED procedure in SAS Version 9.3 TS Level 1 M2) with the treatment group, time and treatment by time point interaction as fixed effects and the animal as a random effect, on log-transformed tick (count +1) data. Back-transformed least squares mean, standard error, 95% confidence interval, and range of data were provided for each treatment and each time point. Treatment group comparisons were done in the event of a significant treatment related effect. The level of significance was set at 5%, all tests were two sided.

## Results

At the start of the study, cats weighed between 2.5 and 4.7 kg. All animals were correctly dosed in the study. One cat in the fluralaner-treated group (group 3) was removed from the study on Day 19 and one cat in the selamectin/sarolaner-treated group (group 2) was removed on Day 84 due to respiratory tract infections. The arithmetic mean tick (*I. ricinus*) counts 48 h post-infestation in the control group ranged from 16.6 to 23.6, indicating adequate tick infestation on all study days. The 48 h tick counts (arithmetic and geometric mean), the acaricidal efficacy, the number of cats with ticks and the comparisons between groups on the various study days are summarised in Table 1. At 24 h post infestation, the thumb tick counts in group 2 and group 3 were significantly (*F*_(2,21)_ = 4.63 to 17.13 and *P* ≤ 0.0481) lower compared to the control cats on days 27, 55, 83 and 90. No administration site observations were recorded for the cats in the control group. The administration site observations for the cats in groups 2 and 3 are listed in Table 2. In group 2, spiking of the hair was observed in all cats at 3 h post treatment and in 1 cat at 24 h, as well as white deposits in two cats 24 h after administration. No additional administration site observations were recorded. In group 3, spiking of the hair, greasiness of the haircoat and white deposits were recorded at all time points after treatment.

**Table 1:** Acaricidal efficacy based on the arithmetic (geometric) mean tick counts for *Ixodes ricinus* as well as the number of cats with ticks at the different time points after treatment

Study day	Group 1^a^		Group 2^b^ (Selamectin/Sarolaner)			Group 3^c^ (Fluralaner)		
	*n*	Mean tick count	*n*	Mean tick count	Percentage efficacy	*n*	Mean tick count	Percentage efficacy
Day 28	8/8	16.6 (16.3)	1/8	0.4 (0.3)^*^	97.6 (98.2)	0/7	0.0 (0.0)^*^	100 (100)
Day 56	8/8	17.4 (16.6)	1/8	0.3 (0.1)^*^	98.3 (99.4)	4/7	3.0 (1.5)^*^	82.8 (91.0)
Day 84	8/8	19.1 (18.4)	1/8	0.3 (0.2)^*^	98.4 (98.9)	6/7	6.3 (3.9)^*^	67.1 (78.8)
Day 91	8/8	23.6 (23.4)	2/7	1.0 (0.5)^*^	95.8 (97.8)	6/7	6.3 (3.0)^*^	73.3 (87.2)

^a^Negative control

^b^Cats were treated topically on Days 0, 30 and 60 with selamectin/sarolaner (Stronghold®Plus)

^c^Cats were treated topically on Day 0 with fluralaner (Bravecto®)

^*^Mean tick counts sign (*P* < 0.05) lower than control group

*n* number of cats with ticks

**Table 2:** Administration site observations in the selamectin/sarolaner-treated animals and in the fluralaner-treated animals at the different time-points after treatment (on Day 0). The number of cats with an administration site observation and the total number of cats in the treatment group is provided

Time after treatment	Group 2 (Selamectin/Sarolaner)			Group 3 (Fluralaner)		
	Spiking/stiff hair	Greasiness	White deposits	Spiking/stiff hair	Greasiness	White deposits
Day 0 (30 min)	0/8	0/8	0/8	0/8	0/8	0/8
Day 0 (3 h)	8/8	0/8	0/8	2/8	8/8	0/8
Day 1 (24 h)	1/8	0/8	2/8	8/8	8/8	0/8
Day 3	0/8	0/8	0/8	6/8	6/8	1/8
Day 5	0/8	0/8	0/8	7/8	0/8	2/8

## Discussion

The current study aimed to evaluate the persistent efficacy of a topical application of selamectin/sarolaner or fluralaner against *I. ricinus,* both administered at their minimum recommended label dose. No adverse reactions to treatment were recorded in the study. As expected for any topical treatment, cosmetic observations at the application site were recorded. Spiking of the hair was observed shortly (3 h) after treatment in all selamectin/sarolaner-treated cats and in one cat by 24 h after treatment. White deposits were observed in two cats 24 h after treatment. In the fluralaner-treated animals, spiking of the hair, as well as greasiness and white deposits were recorded up to 5 days after treatment, potentially due to the larger treatment volumes.

The high and consistent efficacy of selemactin/sarolaner against *I. ricinus* [2, 7, 8] was confirmed in the present study. Three monthly treatments at the minimum recommended label dose of 1.0 mg sarolaner (and 6.0 mg/kg selamectin) resulted in a consistently high efficacy (> 95.8%) for at least 91 days. In contrast, the efficacy of the topical treatment with fluralaner declined from Day 56 onwards. This lower than expected efficacy was not due to a treatment failure in a single cat, as live ticks were present on 4 to 6 out of the 7 fluralaner-treated cats from Day 56 onwards. Apart from its intrinsic lower potency against ticks compared to sarolaner [6], the lower than expected efficacy might be due to the specific pharmacokinetics of fluralaner in cats, with rapid absorption and elimination after topical application. The fluralaner plasma concentration in cats treated at 40.0 mg/kg bodyweight declines more than 10-fold between Tmax and Day 63, further decreasing in the third month after treatment. Although the pK/pD threshold for efficacy of fluralaner is not discussed [9], the fluralaner plasma concentration at less than 10% of the Tmax during the third month after treatment does not suggest an extended plateau in cats required to ensure persistent efficacy.

The label dose for both products is a band dose based on the body weight of the cat (40.0 to 93.75 mg/kg for fluralaner and 1.0 to 2.0 mg/kg for sarolaner). A study design with treatment at the minimum recommended dose might be considered as not representative of all doses applied in the field, yet it does provide insight into the efficacy for those cats that are at the lower end of the dose range. The rapid elimination of fluralaner in cats [9] already resulted in a higher minimum recommended dose compared to dogs (40.0 mg *vs* 25.0 mg per kg bodyweight), and although it was previously concluded that fluralaner treatment at 40.0 mg/kg provides sufficient (> 90%) efficacy against *I. ricinus* for 84 days [10], the results of the present laboratory study indicate that this minimum dose does not guarantee consistent efficacy for the entire treatment period which could result in treatment failure in client-owned cats prescribed the product.

## Conclusion

The high and consistent efficacy of three monthly doses of selamectin/sarolaner at the minimum recommended label dose was confirmed in a three month study. The efficacy of fluralaner at minimum recommended label dose decreased from the second month onwards.

## Abbreviations

pD: Pharmacodynamics
pK: Pharmacokinetics
Tmax: Time to reach maximum drug concentration
